# Integrated Ultrasound Device for Precision Bladder Volume Monitoring via Acoustic Focusing and Machine Learning

**DOI:** 10.1002/advs.202520926

**Published:** 2026-01-20

**Authors:** Long Long Cao, Feng Wen Wang, Jingwei Xue, Fulei Liu, Ming Liang Jin

**Affiliations:** ^1^ The Affiliated Taian City Central Hospital of Qingdao University Qingdao University Taian China; ^2^ School of Automation Qingdao University Qingdao Shandong China

**Keywords:** acoustic focusing, bladder volume monitoring, machine learning algorithms, non‐invasive monitoring, wearable ultrasound devices

## Abstract

Bladder volume monitoring is critical for managing lower urinary tract dysfunctions, yet existing methods remain invasive or operator‐dependent and are unsuitable for continuous use. Here, we present a conformable wearable ultrasound system that combines lens‐assisted acoustic focusing with machine‐learning regression to enable non‐invasive bladder volume estimation, while providing a clear path toward future real‐time implementation. A flexible PZT array integrated with a concave acoustic lens enhances lateral energy concentration and depth selectivity, while a Random Forest model was used to map echo‐derived features to bladder volume estimates. In a pilot study, bladder‐volume estimates generated offline after data collection showed good agreement with a benchtop electrical impedance‐based measurement system, supporting the feasibility of non‐invasive bladder volume estimation. The device was operated using conservative low‐voltage, low‐duty‐cycle excitation settings designed to minimize acoustic exposure and be consistent with diagnostic‐ultrasound safety guidance, and biocompatible, flexible encapsulation is designed to support extended wear. Together with compact packaging and low‐power wireless transmission, these attributes support ambulatory, longitudinal bladder monitoring and offer design insights for future wearable ultrasound systems targeting precise and ultimately continuous physiological monitoring.

## Graphical abstract

We present a conformable wearable ultrasound patch for noninvasive bladder volume monitoring. A flexible PZT array combined with a concave acoustic lens concentrates acoustic energy and improves depth selectivity through the anterior pelvic wall. Echo‐derived features are mapped to bladder volume using machine‐learning regression, demonstrating clinically acceptable agreement with an impedance‐based benchtop reference in a pilot study.

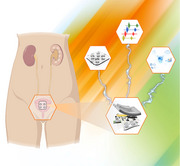

## 1 Introduction

Lower urinary tract dysfunction (LUTD) is a common condition affecting elderly individuals and patients with neurological disorders, typically manifesting as neurogenic bladder, urinary retention, and overactive bladder [1]. These disorders not only significantly reduce patients’ quality of life but may also lead to serious clinical complications such as urinary tract infections, bladder overdistension, and progressive renal impairment [2]. In the diagnosis and management of LUTD, accurate assessment of bladder volume plays a critical clinical role. Conventional methods for bladder volume measurement primarily rely on B‐mode ultrasound, which requires bulky equipment and trained operators, making it unsuitable for long‐term follow‐up or home‐based monitoring [3, 4, 5]. Although intermittent catheterization can indirectly estimate bladder volume by measuring post‐void residual urine, it is invasive and may cause discomfort and increase the risk of infection [6, 7, 8]. These constraints highlight the need for a noninvasive, user‐friendly, and long‐term applicable bladder monitoring technology. Outside formal care settings, bladder status is often inferred from voiding diaries and symptom questionnaires, which provide only indirect and sometimes unreliable information, especially in patients with impaired bladder sensation. Despite their clinical utility, these approaches remain inherently intermittent and cannot capture the dynamic filling and voiding cycles that are critical for diagnosing and managing lower urinary tract dysfunction in daily life.

In recent years, rapid advances in wearable technology have enabled noninvasive, continuous physiological monitoring [9, 10, 11, 12, 13, 14, 15, 16]. Wearable devices designed to capture biological signals from the skin, vasculature, brain, and deep tissues have achieved notable progress in flexible array sensor design, signal‐processing algorithm optimization, and long‐term wearability, offering strategic design frameworks for advancing bladder volume monitoring [11, 17, 18, 19, 20, 21, 22, 23, 24, 25, 26, 27, 28]. At present, wearable devices integrating ultrasound, bioelectrical impedance, or near‐infrared spectroscopy (NIRS) have been applied to bladder volume measurement [29, 30, 31, 32, 33, 34]. Among these, bioelectrical impedance analysis and NIRS are prone to motion artifacts and variability in urine composition, which limit real‐time measurement accuracy [35, 36, 37, 38, 39]. In contrast, ultrasound technology offers superior tissue penetration and detection depth, enabling effective acquisition of echo signals from the bladder region [31, 40, 41, 42, 43, 44, 45, 46, 47, 48]. However, wearable ultrasound technologies face inherent challenges due to the complex anatomy of the anterior pelvic wall. The propagation of ultrasound waves through this multi‐layered and acoustically heterogeneous structure leads to significant beam distortion, scattering, and energy loss, which degrade signal quality. Conventional algorithms, typically based on rigid geometric models like a fixed ellipsoid, cannot accommodate the anatomical variability and poor signal conditions caused by these phenomena, thus limiting measurement accuracy [41, 49]. Therefore, a wearable ultrasound system that can robustly acquire bladder echoes through the anterior pelvic wall and translate them into quantitative bladder‐volume estimates with practical reliability would directly address these unmet clinical needs.

To directly overcome the challenges of acoustic distortion and attenuation, we introduce a wearable bladder monitoring system centered on a lens‐focused ultrasound transducer and an intelligent signal processing framework. A key feature is the integration of a concave acoustic lens with a flexible array, which significantly enhances focusing precision within the bladder region. A flexible PCB with serpentine interconnects is implemented to ensure both long‐term wearing comfort and stable signal acquisition. The hardware demonstrates robust durability and skin conformability, while the software incorporates a machine‐learning‐based regression algorithm to optimize signal processing, thereby improving the accuracy and robustness of bladder volume estimation. Experimental validation involving participants across different age groups confirmed the accuracy and reliability of the system. In addition, the system supports wireless connectivity and low‐latency transmission of echo‐derived data to mobile devices; however, in this pilot study, model‐based bladder‐volume estimates were generated offline after data collection, while the system architecture remains compatible with future real‐time implementation. Through hardware‐software co‐design integrating ultrasound arrays, flexible electronics, and intelligent algorithms, the proposed system provides a design strategy that may guide improvements in focusing precision and algorithm adaptability for wearable ultrasound, while its use of low‐cost materials and streamlined fabrication highlights the potential for an economical, non‐invasive solution that demonstrates clinically acceptable accuracy in preliminary tests. Such capabilities are particularly relevant for home‐based management of patients with lower urinary tract dysfunction, including neurogenic bladder, chronic urinary retention, and overactive bladder, as well as for perioperative and post‐void residual monitoring after urologic or pelvic surgery and for long‐term surveillance in rehabilitation or long‐term care settings where access to conventional ultrasound is limited. Continuous or repeated bladder volume feedback may also benefit individuals with impaired bladder sensation by enabling timely detection of impending overdistension, which may help alleviate sustained bladder pressure and reduce the risk of upper urinary tract deterioration and progressive renal impairment.

## 2 Results and Discussion

### 2.1 Design and Working Principle

**FIGURE 1:**
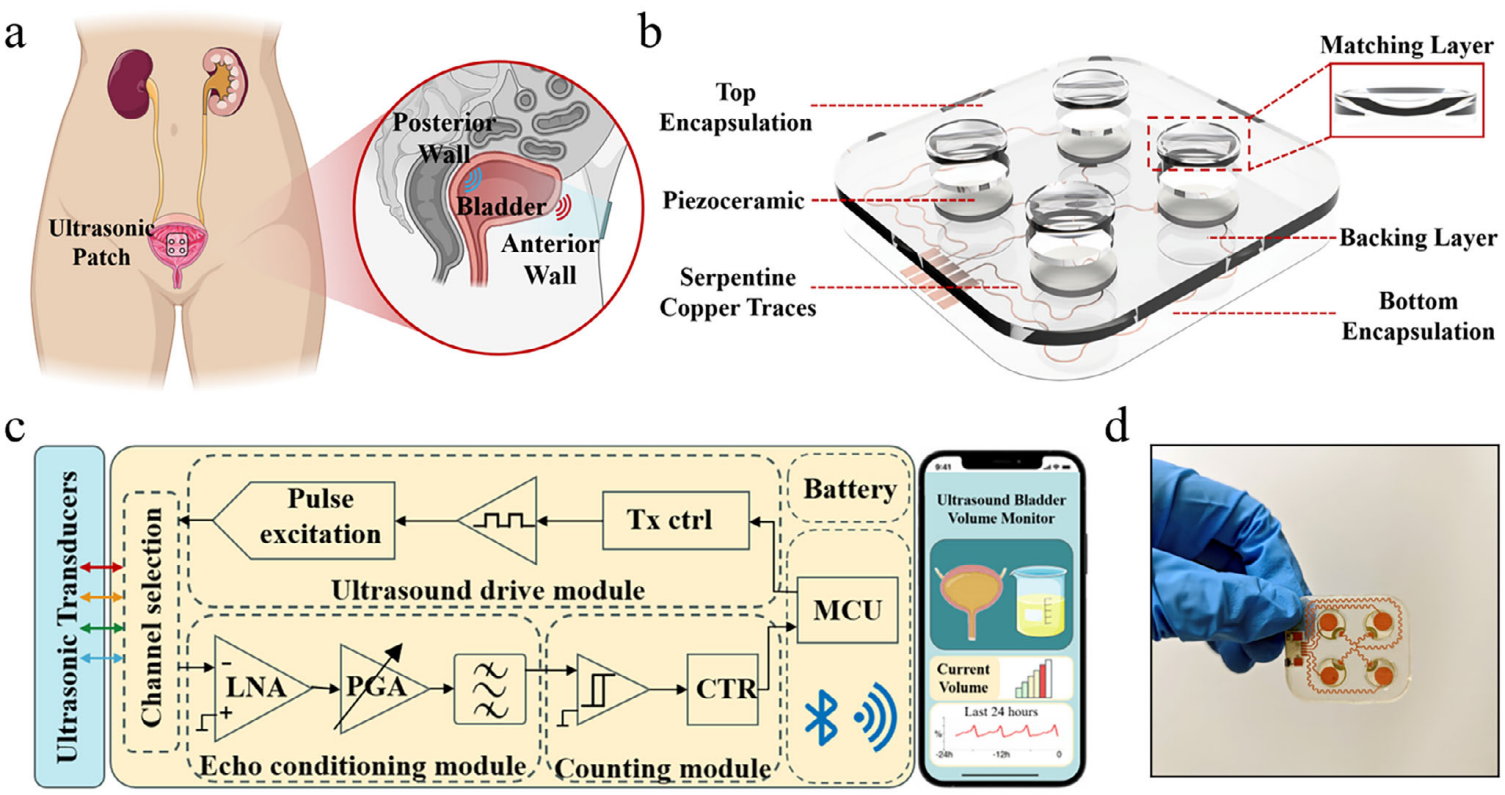
Design and Working Principle of the Wearable Ultrasound for Bladder Volume Monitoring. a) Schematic diagram of the conformal wearable ultrasound system for bladder volume monitoring; b) Disassembly view of the focused ultrasound transducer; c) Design schematic of the wearable device, illustrating the processing and visualization of the driving and echo signals; d) Photograph of the ultrasound transducers.

The proposed wearable bladder monitoring system was developed for wireless and non‐invasive operation while maintaining conformal contact with the abdominal surface for long‐term wearability, with a hardware architecture that is compatible with future real‐time implementation. The system integrates a flexible ultrasound patch, lens‐assisted acoustic focusing, and compact electronics. Each piezoelectric element was designed for operation at a center frequency of 2 MHz, while the 2 × 2 array configuration yielded an aperture well suited for targeting the bladder region of interest. The 2 × 2 array configuration was adopted to strike an optimal balance between spatial sampling capability and overall system complexity. This design provides multiple independent measurement channels, enhancing robustness against acoustic heterogeneity compared to a single element, while avoiding the excessive electronic channels, power consumption, and data processing overhead that would be required by a larger phased array. Figure 1a shows the conceptual schematic of the wearable device. The patch is designed to conform closely to the lower abdomen, enabling stable repeated measurements during daily activities and providing a clear path toward future continuous monitoring. The skin–conformal interface ensures stable acoustic coupling across a wide range of abdominal morphologies, thereby reducing motion artifacts and improving the reliability of the acquired echo signals. The principle of ultrasonic distance measurement, in which the transducer emits a short acoustic pulse that propagates through tissue and is partially reflected at impedance boundaries such as the anterior and posterior bladder walls, is illustrated in Figure S1. The time‐of‐flight (ToF) between the transmitted and received signals directly corresponds to the separation distance of these boundaries, thereby providing the basis for bladder volume estimation.

The structural schematic of the focused ultrasound transducer is presented in Figure 1b. Each element was constructed from a PZT‐5 piezoelectric ceramic disc (10 mm diameter, 1 mm thickness) mounted on a polyimide (PI) flexible substrate. The four elements were arranged in a compact 2 × 2 layout to form a mini‐aperture aligned with the suprapubic window, balancing spatial sampling capability and placement tolerance while maintaining overall mechanical flexibility for wearable use. The PI substrate (approximately 50 µm thick) allowed bending to radii below 10 mm without delamination, and impedance tests during cyclic bending revealed < 5% variation, confirming stable electrical performance under abdominal motion. Serpentine copper interconnects connect each element, providing mechanical stretchability and stable electrical conduction under repeated deformation. The electrical contacts are reinforced with conductive silver epoxy, ensuring low‐resistance bonding. Individual concave acrylic lenses (four in total) were integrated above the transducer elements at the front interface, simultaneously serving as acoustic matching layers and providing element‐wise focusing by shaping the outgoing wavefront toward the bladder region. As shown in Figure S2, the concave acoustic lens reshapes the outgoing wavefront by introducing controlled phase shifts across the transducer aperture, which focuses the acoustic energy into a well‐defined focal region within the pelvic cavity. This focusing mechanism improves lateral resolution, depth selectivity, and the precision of bladder wall detection compared with unfocused planar transducers [50]. While the fixed focal length presents a limitation for extreme anatomical variations, it was optimized for the most common bladder depth range in the target adult population. This design provides a substantial signal‐to‐noise improvement for the majority of users, representing a practical trade‐off between performance and complexity. The complementary use of a multi‐element array further enhances robustness by providing spatial diversity against minor mispositioning. For long‐term monitoring, the lens parameters could be personalized based on an individual's baseline anatomy, a strategy that would further enhance measurement accuracy and advance the potential for personalized care. A thin epoxy resin backing layer further suppresses unwanted reverberations, and the entire unit is encapsulated in a curved housing, which enhances conformity and ensures consistent coupling with the skin.

The signal acquisition and processing pipeline is illustrated in Figure 1c. Excitation pulses are generated by a compact driving circuit and sequentially delivered to each transducer element, which emits ultrasound waves toward the bladder. During operation, ultrasound pulses were driven at a low‐voltage excitation with a pulse repetition frequency selected to ensure both sufficient temporal resolution and compliance with ultrasound safety guidelines. Reflections from internal interfaces, especially from the anterior and posterior bladder walls, are detected by the same transducer elements and then passed through stages of amplification, filtering, and digitization. The propagation distance d was derived from the ToF according to d = (c·Δt)/2, assuming c = 1540 m/s in soft tissue (no temperature compensation was applied in this pilot study). The processed echo signals are subsequently transmitted to a local computational module, where relevant features are extracted and analyzed by a machine‐learning‐based regression algorithm to enable bladder volume estimation; in this pilot study, model‐based predictions were generated offline after data collection. Figure 1d shows the fabricated 2 × 2 transducer array integrated on the PI substrate. The serpentine interconnect layout, shown in Figure S3, highlights the mechanical compliance and robustness of the design, enabling the patch to adapt to abdominal curvature while maintaining electrical stability. The fabrication steps and assembly process of the array are summarized in Figure S4, confirming the feasibility and reproducibility of the design.

In summary, the integration of a skin‐conformal patch, lens‐assisted focused transducers, and flexible serpentine interconnects establishes a compact and reliable platform for wearable ultrasound monitoring. The integration of conformal mechanics and acoustic focusing in this design offers initial improvements compared with existing bladder monitoring devices, thereby laying the groundwork for future designs that aim to achieve accurate, continuous, and user‐friendly monitoring in practical settings.

### 2.2 Characteristics of the Focused Ultrasound Sensors

**FIGURE 2:**
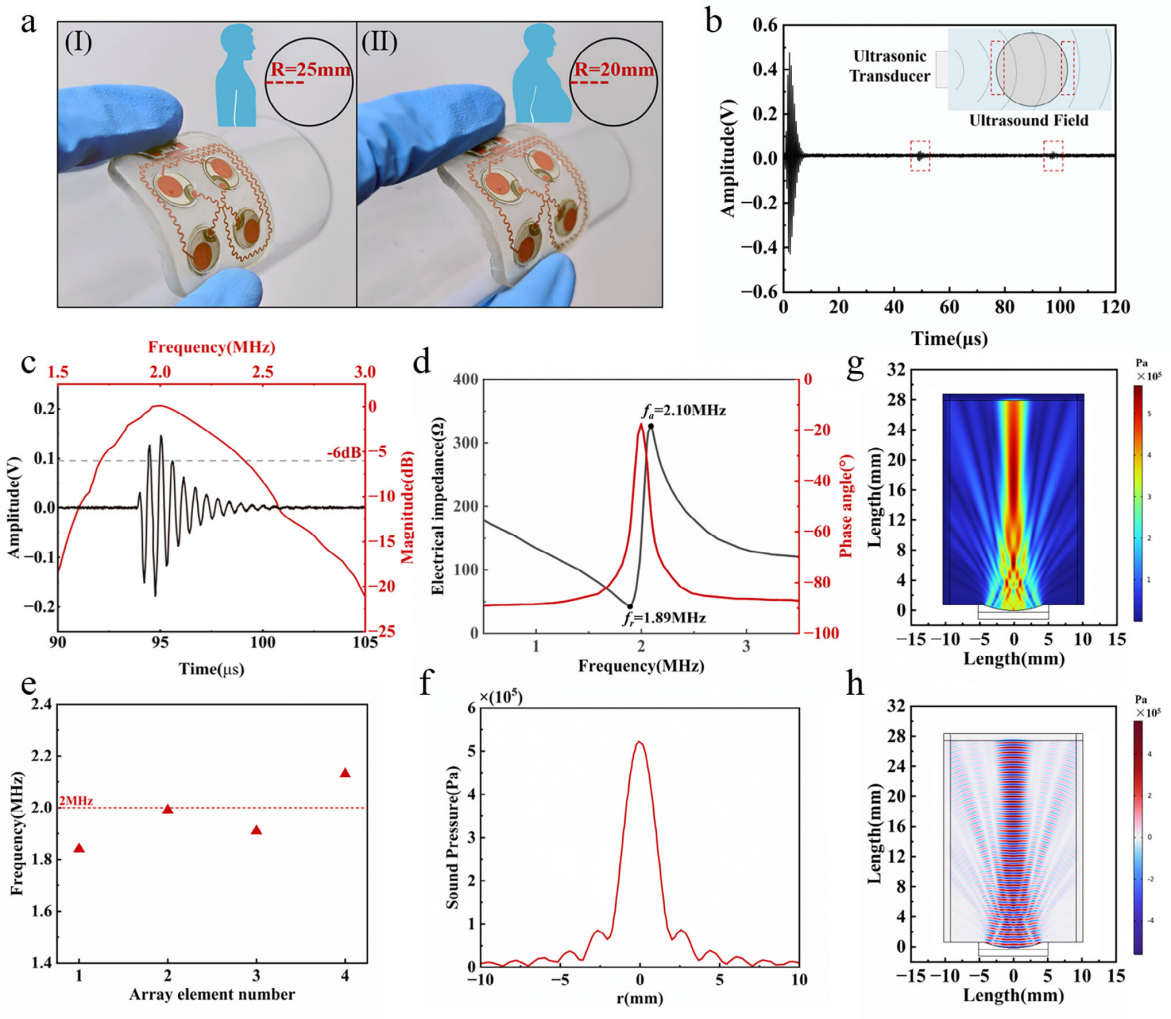
Characteristics of the Focused Ultrasound Sensors. a) Optical image showing the ultrasound sensor attached to surfaces with varying radii, demonstrating its mechanical flexibility; b) Pulse‐echo response of the transducer; c) Pulse‐echo response and spectrum of the transducer; d) Measurement of the impedance and phase angle spectrum of the sensor; e) Center frequencies of the four transducers. Measurements were obtained from the 2 × 2 array (n = 4 elements); f) Sound pressure distribution in the vertical direction; g) Simulation of the acoustic pressure distribution produced by the transducer's acoustic lens; h) Simulation of the focusing effect of the acoustic lens and the total sound pressure distribution.

To rigorously evaluate the performance of the fabricated focused ultrasound transducers, a series of structural, electrical, and acoustic characterizations were carried out. These assessments encompassed flexibility on curved substrates, pulse–echo behavior and spectral response in water, impedance and resonance stability across array elements, and both experimental and simulated analyses of beam focusing. The cross‐section of a single ultrasonic transducer is shown in Figure S5. Collectively, the results demonstrate reliable mechanical compliance with nonplanar anatomical surfaces, stable electromechanical coupling near the design frequency of 2 MHz, and effective lens‐assisted acoustic focusing that achieves depth‐selective interrogation of bladder walls. Figure 2a shows the mechanical flexibility test of the transducer array, performed by laminating the device onto cylindrical substrates with radii of 25 and 20 mm. These radii were selected to approximate the abdominal curvature typically observed in adult subjects, thereby simulating realistic conditions for a wearable application. The array maintained continuous contact with both substrates without delamination or electrical discontinuity, confirming that the polyimide substrate and serpentine copper interconnects provide sufficient compliance to accommodate inter‐subject morphological variability. Such conformal integration is critical for wearable operation, as it ensures stable acoustic coupling during body movement and minimizes motion‐induced artifacts during long‐term monitoring.

The pulse–echo response of the focused transducer, measured in a water‐tank setup with a planar glass reflector, is shown in Figure 2b. Two distinct echoes corresponding to the front and back reflecting surfaces can be clearly identified as temporally separated peaks in the time‐domain signal. This separation demonstrates that the emitted pulse is sufficiently short and the device provides adequate axial resolution to resolve multilayer interfaces. Such capability is critical for bladder monitoring, where the anterior and posterior bladder walls must be distinguished to enable accurate volume estimation. If the pulse duration were longer or the bandwidth narrower, these echoes would overlap, leading to significant errors in cavity size reconstruction. The observed pulse–echo characteristics therefore validate the transducer's suitability for depth‐resolved interrogation of the bladder cavity. Figure 2c further presents the frequency‐domain spectrum of the same signal, with a center frequency near 2 MHz and a −6 dB bandwidth of approximately 25%. The choice of 2 MHz as the operating frequency reflects a balance between sufficient penetration through abdominal tissue and spatial resolution for delineating bladder walls. The −6 dB criterion was adopted, as it provides a more accurate representation of the dominant frequency components underlying pulse formation, corresponding to the spectral range where the normalized amplitude remains at least 50% of its peak. This definition provides a more representative measure of the effective acoustic energy distribution relevant to pulse compactness and echo separation, whereas the conventional −3 dB bandwidth may underestimate the practical frequency content of the emitted pulse. A fractional bandwidth of approximately 25% ensures that the emitted pulses are temporally compact, directly supporting reliable separation of echoes from the anterior and posterior bladder walls [51]. Together, the time‐domain and frequency‐domain results confirm that the focused transducer provides both the resolution and frequency response necessary for bladder wall detection under wearable conditions.

Figure 2d presents the electrical impedance and phase spectra of the focused transducer. A pronounced resonance was observed at 1.89 MHz and an anti‐resonance peak at 2.10 MHz, consistent with the acoustic center frequency identified in the pulse–echo measurements. The clear resonance–anti‐resonance pair confirms stable electromechanical coupling of the PZT‐5 elements, indicating that the device efficiently converts electrical excitation into acoustic energy in the targeted frequency range. The center frequency distribution of the four elements in the 2 × 2 array is further summarized in Figure 2e. All elements exhibited resonance values clustered around 2.0 MHz, with deviations less than ±0.15 MHz. Such narrow dispersion provides uniform element sensitivity, thereby enabling stable multi‐element performance and enhancing the reliability of downstream machine‐learning algorithms for bladder volume estimation.

The efficacy of the acoustic lens in focusing energy at a specified focal region is demonstrated through both experimental and simulation results. Figure 2f shows the measured radial sound pressure distribution at the focal point, where a sharp peak is observed at the center, indicating highly localized energy concentration. This result confirms that the lens‐assisted transducer delivers focused acoustic energy at the intended depth within the pelvic cavity, minimizing the lateral dispersion of sound waves. The narrow central peak of the pressure profile further supports the lens's capability to enhance focal resolution, achieving a high pressure at the target area with minimal side lobes. For a quantitative and intuitive comparison of the focusing performance, the acoustic field of the lens‐assisted transducer was contrasted with that of an otherwise identical planar transducer, as shown in Figure S6. In the simulation, Figure 2g presents the 2D acoustic pressure field produced by the transducer's lens, demonstrating effective lateral and axial confinement of the ultrasound energy. The simulation shows a well‐defined focal region, where the energy is tightly focused along the axial direction, which is crucial for improving the signal‐to‐noise ratio of bladder‐wall echoes. This focused energy distribution is key to distinguishing between the anterior and posterior bladder walls, a critical factor for accurate bladder volume estimation. Finally, Figure 2h displays the total sound pressure distribution simulation, showing the overall beam shape and focal characteristics. The simulation results confirmed that the acoustic lens effectively shapes the ultrasound beam, concentrating energy at the target depth and thereby enhancing focus on the bladder region. Compared with planar elements of identical aperture, the lens‐assisted configuration reduced the −6 dB lateral beamwidth by approximately 40%–60% and suppressed side‐lobe levels by 6–10 dB. In absolute terms, the focused beam exhibited a −6 dB lateral width of 3–5 mm and an axial focal zone of 8–12 mm, demonstrating improved lateral resolution and depth selectivity relative to unfocused elements. This degree of focusing is advantageous for wearable bladder monitoring, as it improves the signal‐to‐noise ratio of bladder‐wall echoes without requiring high channel counts or complex phased‐array electronics. The consistency between the simulated and observed pressure distributions confirms that the transducer and lens design achieve the intended acoustic performance, ensuring high‐quality, depth‐resolved bladder monitoring.

Collectively, the comprehensive mechanical, electrical, and acoustic characterizations establish that the focused transducer array exhibits robust conformity to curved surfaces, stable and reproducible electromechanical behavior around 2 MHz, and effective lens‐assisted focusing validated by both experiment and simulation, which facilitates more reliable detection of anterior and posterior bladder wall echoes and is essential for accurate bladder volume estimation in wearable settings. These features form a solid foundation for reliable bladder‐wall interrogation and subsequent integration into the wearable monitoring system.

### 2.3 Design and Communication of the Wearable Ultrasound System

To enable practical bladder monitoring, the focused ultrasound transducers were integrated into a fully packaged wearable platform that combines miniaturized hardware, flexible encapsulation, and streamlined signal communication. The system was designed with three principal requirements, which include compactness to ensure comfort and unobtrusiveness during daily activities, mechanical robustness to maintain stable operation under body motion and skin deformation, and efficient signal flow to reliably transmit acoustic information from the transducers to external computational units. The overall architecture incorporates biocompatible packaging materials, mechanically compliant interconnects, low‐noise driving and receiving circuitry, and wireless transmission modules, thereby establishing a hardware foundation for continuous, real‐time bladder volume monitoring in realistic wearable conditions.

**FIGURE 3:**
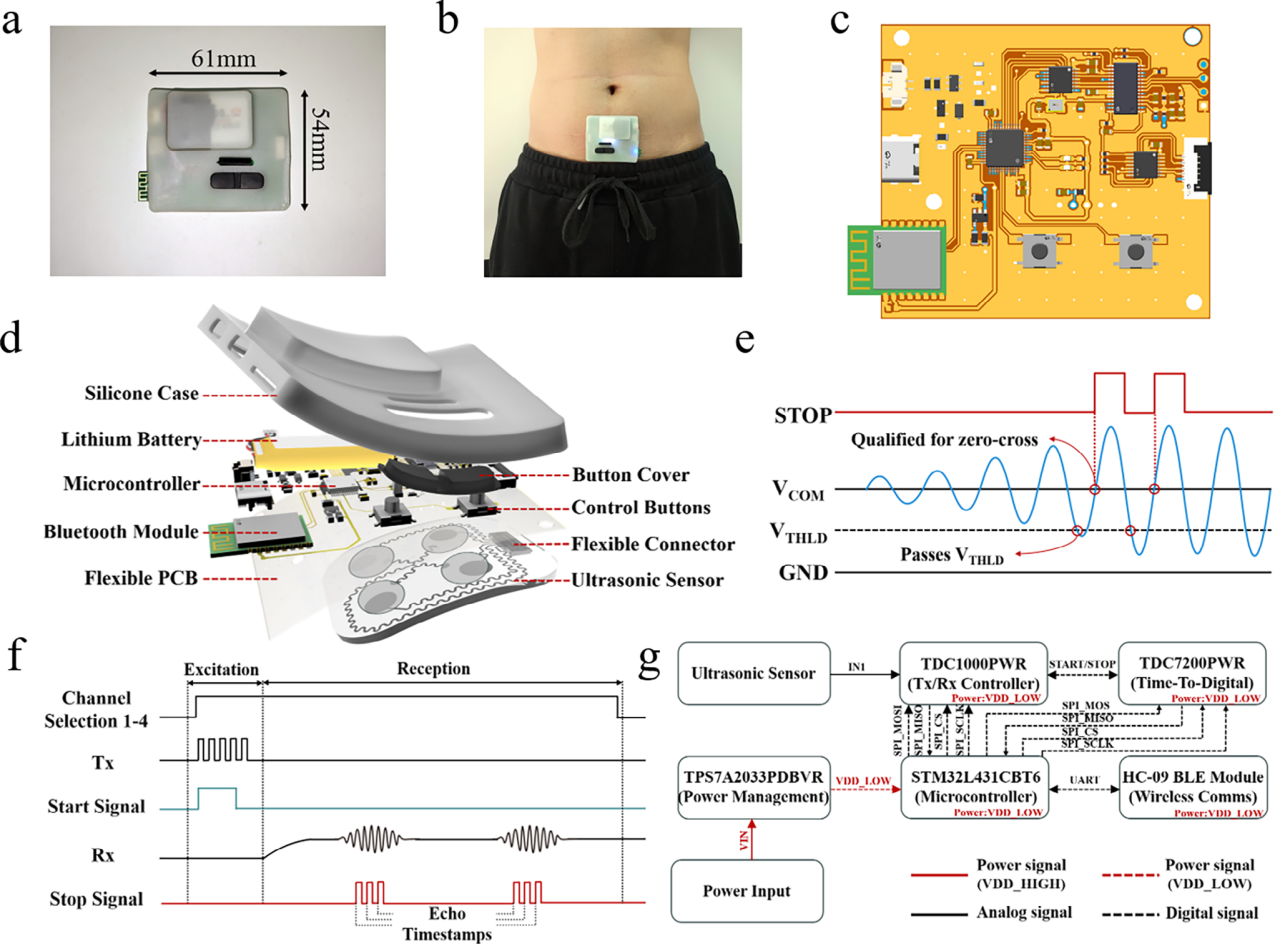
Design and Communication of the Wearable Ultrasound for Bladder Monitoring. a) Images of the packaged wearable devices with their dimensions; b) The packaged wearable ultrasound device placed on the abdomen for bladder volume monitoring; c) Flexible circuit board used for driving ultrasonic patches; d) Packaging of the electronic components and their connection to the ultrasound sensors; e) Conversion process from sound wave signals to voltage signals; f) Timing diagram of the wearable device; g) Functional block diagram of the signal chain for the wearable device.

Figure 3a shows the packaged wearable ultrasound device with overall dimensions of approximately 61 mm × 54 mm. The device has a maximum thickness of approximately 12 mm, comparable to other wearable health monitors such as continuous glucose sensors. The compact rectangular footprint minimizes bulkiness while providing sufficient internal volume to accommodate the driving electronics, signal conditioning circuits, and wireless transmission module. The rechargeable lithium battery supports continuous operation for many hours under typical measurement duty cycles (approximately 8–12 h), enabling overnight or day‐long bladder monitoring without the need for recharging. Under the operating conditions used in this study, the measured current profile corresponds to an average system power on the order of tens of milliwatts, which is comparable to other skin‐mounted wearable devices and sufficient to support continuous or ambulatory bladder monitoring. The housing is made from a lightweight, biocompatible polymer, designed to ensure comfort during prolonged skin contact. Although the exact mass may vary with battery capacity, the lightweight construction combined with rounded edges and smooth surfaces minimizes mechanical irritation and allows the device to be worn unobtrusively under clothing. The device is placed on the lower abdomen for bladder monitoring, as demonstrated in Figure 3b. The moderate size and thin profile allow the system to conform to the abdominal contour, while the integrated fastening mechanism maintains stable attachment during motion. This ergonomic design ensures consistent acoustic coupling to the skin and supports long‐term, user‐friendly operation in daily monitoring scenarios.

Figure 3c presents a photograph of the fabricated flexible printed circuit board designed to drive and interface with the ultrasound transducer array. The board features a compact layout that integrates the ultrasonic front‐end (TDC1000/TDC7200) and the STM32L431 microcontroller on a polyimide substrate. The packaging architecture of the wearable device, highlighting the integration of electronic components with the ultrasound sensors, is illustrated in Figure 3d. A biocompatible silicone case serves as the outer enclosure, providing mechanical protection, environmental sealing, and skin‐safe contact. A rechargeable lithium battery supplies power for extended operation, while a low‐power microcontroller coordinates the excitation, acquisition, and wireless transmission processes. Control buttons and a protective cover allow simple user interaction while preventing accidental activation. The electronics are mounted on a flexible printed circuit board (PCB), which is mechanically linked to the ultrasound sensor array through flexible connectors and serpentine interconnects. The ultrasonic transducers are connected to the main electronic board via flat flexible cables (FFC), enabling reliable signal routing while simultaneously accommodating mechanical deformation during wear. This packaging strategy ensures robust electrical connectivity and mechanical compliance, allowing the system to maintain reliable operation under bending and stretching in daily use. Additional details regarding the hardware implementation, including the PCB layout and circuit schematic, are provided in Figures S7–S10.

Figure 3e details the conversion of received echoes into voltage signals suitable for digital timing. The sinusoidal echo waveform is referenced to a common‐mode voltage (VCOM) for baseline stabilization. A programmable threshold voltage (VTHLD) is then applied to suppress sub‐threshold noise and spurious fluctuations. When the waveform crosses this threshold, the signal is qualified for further processing. A zero‐crossing detector identifies precise points where the waveform crosses the baseline, generating discrete logic pulses. These pulses are translated into a STOP signal, which is used by the time‐to‐digital conversion (TDC) circuitry to mark the exact arrival time of the echo. GND provides the system reference potential for both analog and digital domains. The combination of threshold qualification and zero‐crossing detection ensures robust noise rejection, accurate timing extraction, and hardware‐efficient implementation, thereby enabling low‐power, real‐time echo detection in the wearable system.

The timing diagram of the wearable ultrasound system, which governs the sequence of excitation, echo reception, and digital timestamp generation, is presented in Figure 3f. A channel selection signal enables sequential activation of individual transducer elements within the 2 × 2 array, ensuring that each operates independently without crosstalk. The transmit signal (Tx) triggers the driving circuit to generate short excitation pulses, while a start signal is simultaneously issued to the time‐to‐digital conversion (TDC) circuitry to initiate timing. The received echoes (Rx) are subsequently processed through the analog front‐end, and once qualified by threshold and zero‐crossing detection, corresponding stop signals are generated. These stop signals encode the exact arrival times of the echoes, allowing precise calculation of time‐of‐flight and, consequently, bladder wall separations. This timing scheme ensures strict synchronization between transmission and reception, minimizes overlap between channels, and provides high temporal accuracy for volume estimation. The functional block diagram of the wearable system, summarizing the signal flow from ultrasound generation to wireless transmission, is depicted in Figure 3g. The ultrasound sensors are driven and interrogated by the TDC1000 chip, which functions as a transmit/receive controller and provides analog front‐end conditioning including excitation, amplification, and echo detection. The qualified timing signals are then passed to the TDC7200 time‐to‐digital converter, which measures the precise time‐of‐flight intervals with sub‐nanosecond resolution. These digital timestamps are processed by a low‐power STM32L431 microcontroller, which performs control sequencing, data formatting, and packetization for wireless transmission. To interface the 2 × 2 array, we employed a 4‐channel analog switch (TS3A4751PWR) as a time‐division multiplexer. The four PZT elements were connected to the COM1–COM4 terminals of the switch, whereas the NO1–NO4 terminals were tied together and connected to the TX/RX node of the TDC1000 through series resistors. General‐purpose I/O lines from the STM32L431 microcontroller selected one channel at a time so that, during each firing cycle, only a single element was driven and its echo was routed back to the TDC1000. By sequentially cycling through the four channels, the microcontroller acquired four independent echo measurements per acquisition frame while minimizing electrical crosstalk. For wireless connectivity, the processed data are transmitted via an HC‐09 Bluetooth Low Energy (BLE) module, enabling low‐latency wireless communication with external devices such as smartphones or computers. In this prototype, the BLE link operates over a short range in a controlled environment, and only de‐identified measurement IDs and numerical features are transmitted. Standard BLE pairing was used for establishing the connection, and all received data were stored locally on password‐protected devices without cloud synchronization. A comprehensive security assessment and additional application‐layer protections will be implemented in future versions intended for broader or remote deployment. Under bench testing conditions, the BLE link operated on the LE 1 M PHY and reliably transmitted the per‐frame feature packets with a mean end‐to‐end latency < 50 ms, indicating feasibility for low‐latency wireless data transfer and future real‐time processing. Power regulation for the entire system is provided by the TPS7A2033 low‐dropout regulator, which ensures a stable supply voltage to both analog and digital domains. The integration of these components establishes a streamlined signal chain that is compact, energy‐efficient, and fully compatible with wearable applications, thereby enabling continuous and reliable bladder volume monitoring. In addition to the main system diagram, the underlying communication and power management strategies were also characterized. The SPI communication timing diagram of the ultrasonic echo conditioning module is provided in Figure S11, illustrating the synchronization between the TDCs and the microcontroller during data transfer. This digital protocol ensures reliable and low‐latency communication while minimizing power consumption, which is crucial for wearable operation. The PCB layout highlights the compact arrangement of analog and digital circuits, with careful separation of power and ground planes to reduce electromagnetic interference. Together, these design considerations provide a robust electronic backbone for the wearable ultrasound system, complementing the compact packaging and signal processing chain described above.

Collectively, the packaging design, electronic integration, signal communication, and power management strategies establish a compact and energy‐efficient wearable platform for bladder monitoring. The coordinated hardware modules, supported by robust SPI communication and optimized PCB‐level power distribution, ensure stable operation under realistic wearable conditions. These engineering advances form the technological backbone that enables continuous and user‐friendly data acquisition with real‐time wireless data‐transfer capability. In this pilot study, volume inference was performed offline after data collection.

### 2.4 Machine Learning Based Bladder Volume Estimation and Validation

**FIGURE 4:**
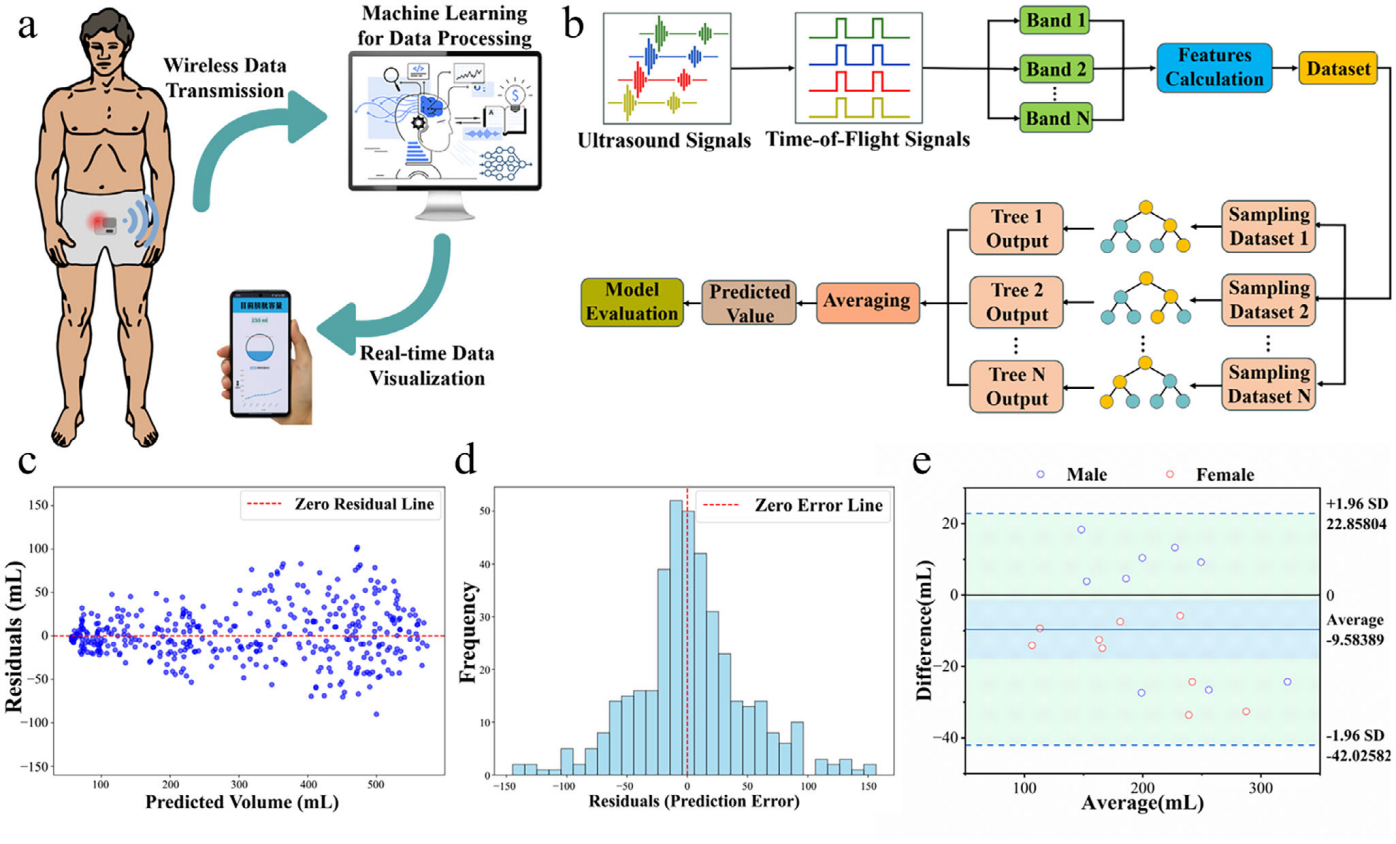
Machine Learning Based Bladder Volume Estimation and Validation. a) Overview of the experimental process of the wearable device, from patient wear to local data storage and post hoc visualization on a nearby tablet/laptop; b) Workflow from the collection of ultrasound signals to the final prediction results; c) Scatter Plot of Residuals against Predicted Volume with Zero Residual Reference Line. Residuals are plotted for n = 360 window‐level predictions; d) Histogram of Residuals with Zero Error Reference Line. The histogram summarizes n = 360 window‐level residuals; e) Bland–Altman Plot Comparing the Wearable Ultrasound Device and the impedance‐based reference device. Bland–Altman analysis was performed on n = 120 acquisition‐level paired estimates, where each wearable estimate was obtained by averaging the three window‐level predictions from the same acquisition.

To demonstrate the practical capability of the wearable ultrasound device, machine learning (ML) methods were employed to estimate bladder volume from acquired ultrasound signals. The objective was to establish a fully automated workflow, in which raw echo data are converted into quantitative predictions of bladder volume and subsequently validated against a benchtop electrical impedance‐based measurement system. The validation strategy focused on residual analysis, error distribution, and agreement testing, thereby providing a comprehensive assessment of both prediction accuracy and clinical reliability. Figure 4a provides an overview of the experimental workflow of the wearable bladder monitoring system, from patient use to data visualization. Once attached to the lower abdomen, the wearable ultrasound device repeatedly acquires echo signals from the bladder region at scheduled time points. A machine learning algorithm is employed to process the incoming acoustic data, extracting key features and mapping them to bladder volume estimates. Echo signals were transmitted via the BLE link to a nearby tablet or laptop for local storage without cloud‐based transmission. In this pilot study, the wearable system did not display real‐time volume estimates during data collection; instead, feature extraction and model‐based volume predictions were generated offline after the study, and all data were stored on password‐protected devices. Together, this end‐to‐end workflow integrates acquisition, wireless data transfer, offline algorithmic inference performed after the study, and post hoc visualization. It demonstrates the practicality of the system for real‐world applications and highlights its potential for future real‐time integration into daily healthcare routines.

Figure 4b outlines the machine‐learning workflow for bladder volume estimation. The model was trained and evaluated on a dataset comprising 120 paired measurements from six healthy volunteers, with comparative reference volume estimates provided by a benchtop electrical impedance‐based measurement system. To increase the number of training samples while preserving the original acquisition protocol, each recorded acquisition frame (a fixed‐duration echo trace) was partitioned into three consecutive, non‐overlapping windows of equal length spanning the full trace from start to end. Each window inherited the same reference volume label measured at that acquisition time point using the benchtop electrical impedance‐based measurement system. Therefore, 120 paired acquisitions yielded 360 window‐level samples that were used as inputs to the machine‐learning models. A leave‐one‐subject‐out cross‐validation (LOSO) strategy was applied, in which data from one participant were held out for testing while the remaining participants’ data were used for training. In leave‐one‐subject‐out cross‐validation, all windows derived from a given participant were assigned exclusively to either the training set or the held‐out test set within each fold, thereby preventing information leakage across folds. Consequently, the three windows originating from any single acquisition frame were always kept together within the same split and were never separated across training and test sets. Direct volume estimation from a single ToF is not feasible because it only provides a 1D distance. Bladder volume is a 3D property, and a single measurement cannot capture the complex shape variations across individuals and filling states. Our multi‐element array, combined with machine learning, is essential to infer the volume from a richer set of spatial and signal features. After echo acquisition, raw signals were converted into ToF traces corresponding to reflections from the anterior and posterior bladder walls. In addition, amplitude ratios, envelope energies in selected frequency bands, and simple morphology descriptors of the echo profile were extracted. These features reflect both propagation delays and the relative strength and stability of bladder wall reflections. All features were standardized to zero mean and unit variance prior to model training to avoid scale bias. A Random Forest regressor was selected as the primary model because of its robustness against outliers and its ability to capture non‐linear feature–volume relationships. Compared with deep neural networks, this ensemble method also reduces computational burden and enhances interpretability, both of which are essential for deployment in wearable systems with limited resources. Each tree was trained on a bootstrap sample of the training set with randomized feature subsets, and predictions were averaged across the ensemble. This strategy ensured that training and test data came from different individuals, thereby preventing information leakage and providing a conservative estimate of generalization performance. Model performance was further assessed using bootstrap resampling to obtain 95% confidence intervals for the mean absolute error (MAE), root mean square error (RMSE), and the coefficient of determination (R^2^). We compared three regression models (linear regression, random forest, and XGBoost) under the same training and testing protocol. As summarized in Figure S12, the random forest regressor achieved the lowest RMSE (48.78) and MAE (35.09), together with the highest coefficient of determination R^2^ (0.9202), and was therefore selected as the final model for bladder volume estimation. Residual diagnostics suggested approximate normality and no strong heteroscedasticity. As shown in Figure 4c, residuals were symmetrically distributed around zero without clear systematic bias, although variance increased slightly at higher filling levels, likely due to greater anatomical variability and acoustic attenuation. The histogram of residuals in Figure 4d indicates that the majority of predictions fell within clinically acceptable limits when compared with reference measurements. From a clinical perspective, error tolerances on the order of tens of milliliters may be acceptable for certain bladder‐management decisions, although the acceptable error depends on the specific clinical context and decision thresholds. Compared with conventional geometric fitting approaches that assume a fixed ellipsoidal bladder shape, the ensemble regression framework demonstrated improved adaptability to inter‐subject differences in bladder morphology and filling patterns [31]. These findings suggest that machine‐learning‐based regression, when coupled with lens‐assisted focusing, can provide a reliable foundation for continuous bladder monitoring in daily life.

To assess agreement between the wearable ultrasound estimates and the impedance‐based reference device, a Bland–Altman (B–A) analysis was performed in Figure 4e. Unlike correlation coefficients, which quantify association, Bland–Altman analysis directly characterizes the mean bias and dispersion of paired differences, providing an interpretable measure of agreement between two measurement methods [52]. In our Bland–Altman analysis (n = 120 acquisition‐level pairs; each wearable estimate was obtained by averaging the three window‐level predictions from the same acquisition), the mean difference was approximately –9.6 mL, indicating a small negative bias of the wearable estimates relative to the impedance‐based reference device. The 95% limits of agreement (LoA) ranged from −42.0 to +22.9 mL, with most data points falling within this interval. Regression of the differences against the mean values suggested no obvious proportional bias across the evaluated filling range. Because the reference standard in this pilot study was the impedance‐based reference device, these results should be interpreted as a feasibility‐level benchmark of method agreement. Future studies will validate the wearable system against standard‐of‐care bladder‐volume assessments in larger and more diverse cohorts.

In summary, the present analyses show that integrating machine‐learning regression with lens‐assisted ultrasound sensing enables volume estimation that is both statistically consistent and clinically acceptable. The analyses highlight minimal bias and stable performance across different participants, underscoring the methodological reliability of the proposed framework for wearable bladder monitoring.

## 3 Conclusion

This work demonstrates a flexible and wearable ultrasound platform that integrates conformal mechanics, lens‐assisted focusing, and machine‐learning regression to achieve reliable bladder volume estimation. Mechanical and acoustic characterizations confirmed stable conformity on curved surfaces, reproducible electromechanical coupling near 2 MHz, and effective lens‐guided focusing, enabling depth‐selective interrogation of bladder walls. The system further incorporates compact electronics, wireless transmission, and automated data processing, resulting in a streamlined workflow from signal acquisition to volume prediction. By leveraging low‐cost components and simplified fabrication processes, the platform also offers a cost‐effective solution suitable for large‐scale deployment. Validation against a benchtop electrical impedance‐based reference system showed promising agreement and robustness to inter‐subject variability in this pilot study. The present pilot study was limited by a relatively small sample size and a cohort of healthy volunteers, and future work will involve larger and more diverse patient populations to further validate the robustness and generalizability of the proposed system.

Looking forward, clinical translation of this system holds promise for enabling non‐invasive, continuous bladder monitoring that complements established diagnostic pathways. Its design supports user‐friendly operation and offers a clear path toward real‐time implementation; however, in this pilot study, model‐based volume estimates were generated offline after data collection. Furthermore, integration with wireless data infrastructures positions the platform for seamless incorporation into emerging digital health ecosystems. Beyond immediate applications, the design paradigm introduced here may inform the broader development of wearable ultrasound technologies, providing conceptual guidance for the design of systems aimed at precise and continuous physiological monitoring. Collectively, this work advances wearable ultrasound from laboratory feasibility toward practical clinical implementation, while offering a reference point for next‐generation biomedical sensing platforms.

## 4 Experimental Section

### 4.1 Materials

The piezoelectric elements used for the transducer array were single‐sided electrode PZT‐5 ceramics (1 mm thickness, 10 mm diameter; Shenzhen Keyi Sheng Electronics, China). The flexible substrate was polyimide (PI, thickness approximately 50 µm), chosen for its mechanical compliance and thermal stability. Encapsulation was achieved with polydimethylsiloxane (PDMS; Dow Corning, USA), while the outer housing employed medical‐grade silicone rubber to ensure biocompatibility and long‐term skin contact. Electrical connections between elements were reinforced using conductive silver epoxy (3022 E‐Solder; Von Roll, Switzerland). The acoustic lens was fabricated from acrylic (custom‐processed; Yalangdiao, Shanghai, China) to provide controlled focusing. All electronic components, including the driving, receiving, and wireless modules, were sourced from commercial suppliers (Jialichuang, China). PDMS and medical‐grade silicone rubber are widely used as encapsulation and skin‐contact materials in wearable and implantable biomedical devices, and previous studies have reported good skin tolerance and low cytotoxicity under conditions of long‐term wear. In this pilot study, no additional in vitro cytotoxicity assays were performed on the assembled device; however, the patch was worn on the lower abdomen for continuous periods of up to several hours, and no skin irritation, redness, or discomfort was reported by any participant. Formal ISO 10993 biocompatibility and skin‐irritation testing will be required in future work prior to extended clinical deployment [53, 54, 55].

### 4.2 Fabrication of Flexible Ultrasound Transducer Array

The flexible ultrasound transducer array was fabricated through a multi‐step process. First, a serpentine copper interconnect pattern was laser‐etched on a polyimide substrate to form the flexible circuit architecture. Conductive silver epoxy (3022 E‐Solder, Von Roll, Switzerland) was then applied to bond the PZT‐5 ceramic discs to the patterned circuit, ensuring both mechanical adhesion and low‐resistance electrical contact. To improve acoustic impedance matching and achieve beam focusing, a concave acrylic acoustic lens (organic glass, custom‐fabricated) was precisely aligned and mounted on the front surface of the array. A thin epoxy resin backing layer was applied to the rear surface of each element to attenuate backward‐propagating waves and reduce reverberations. Finally, the assembled components were encapsulated to yield a flexible and mechanically robust array capable of efficient ultrasound transmission and reception. Stepwise fabrication procedures and photographs of the completed array are provided in Figure S4.

### 4.3 Characteristics of Ultrasound Transducers

The electrical and acoustic characteristics of the fabricated ultrasound transducers were systematically evaluated. All acoustic measurements were conducted in a deionized water tank to provide a controlled propagation medium. The water temperature was maintained at room temperature (22°C–25°C) to ensure acoustic property consistency. Electrical parameters, including impedance, phase, and conductance spectra, were measured using an impedance analyzer (4294A, Agilent, USA) to determine resonance and anti‐resonance behavior. For acoustic performance assessment, the pulse–echo response was recorded by exciting the transducers with an arbitrary waveform generator (33250A, Agilent, USA) and capturing reflected echoes from a planar glass reflector using a digital oscilloscope (DSOX1102A, Keysight, USA). This setup enabled quantitative analysis of both the time‐domain response, including echo separation and pulse duration, and the frequency‐domain response derived from the Fourier transform of the recorded signals. These characterizations collectively provided insight into the transducer's resonance stability, bandwidth, and suitability for bladder‐wall interrogation.

### 4.4 Finite Element Analysis (FEA)

The acoustic performance of the transducer was evaluated through finite element simulations conducted in COMSOL Multiphysics (COMSOL, Inc., USA). The Multiphysics model coupled electrostatics, solid mechanics, piezoelectricity, and frequency‐domain pressure acoustics, incorporating acoustic–structural interfaces to capture wave propagation across material boundaries. Material properties of PZT‐5, polyimide, epoxy, and acrylic were assigned based on manufacturer datasheets and validated against experimental impedance and pulse–echo measurements. The computational domain was surrounded by perfectly matched layers (PMLs) to minimize boundary reflections, and the mesh size was selected to be less than one‐sixth of the acoustic wavelength to ensure numerical accuracy. Simulations were performed at the operating frequency of 2 MHz. The results demonstrated a well‐defined focal spot located 20–30 mm beneath the transducer surface, with a −6 dB lateral beamwidth of approximately 3–5 mm and side‐lobe suppression of approximately 6–10 dB compared with a planar element. These findings confirm that the acrylic acoustic lens effectively concentrates acoustic energy within the bladder region of interest, thereby enhancing lateral resolution and depth selectivity for bladder‐wall interrogation.

### 4.5 Design and Fabrication of the Electronic System

The electronic system was designed to achieve compact integration, low‐power operation, and reliable wireless communication for wearable use. The core processing unit was a low‐power microcontroller (STM32L431CBT6, STMicroelectronics), which coordinated ultrasound excitation, echo acquisition, and data handling; Bluetooth Low Energy (BLE) communication for wireless data transmission was implemented via an external module (HC‐09). Feature extraction and model‐based inference were performed on the paired tablet/laptop in this study.

An integrated ultrasonic front‐end (TDC1000PWR, Texas Instruments) was employed to both excite and receive signals from the flexible PZT array. The TDC1000 generated short excitation pulses on its TX pins to drive the selected transducer element and used its built‐in programmable gain and comparator circuitry to detect returning echoes, thereby reducing the need for discrete pulser and amplifier components. To interface the 2 × 2 array, a 4‐channel analog switch (TS3A4751PWR, Texas Instruments) was used to sequentially connect each transducer element to the TDC1000. The switch was controlled by GPIO lines from the STM32L431, enabling time‐division multiplexing of the four elements while minimizing crosstalk and preserving signal integrity. Precise time‐of‐flight information was obtained using an external time‐to‐digital converter (TDC7200PWR, Texas Instruments), which recorded the interval between the excitation trigger and the echo detection signal from the TDC1000 with sub‐nanosecond resolution. All components were mounted on a flexible printed circuit board (PCB), with careful routing and separation of analog and digital domains to reduce electromagnetic interference and facilitate conformal skin contact. The system was powered by a rechargeable lithium battery managed by a linear charger (TP4057, Top Power ASIC) and regulated by a low‐dropout regulator (TPS7A2033, Texas Instruments), supporting extended operation under wearable conditions.

To contextualize the prototype with respect to established ultrasound safety guidance, the wearable transducer was operated at a center frequency of approximately 2.0 MHz using low‐voltage, short excitation pulses with a low duty cycle to minimize acoustic exposure. Because a calibrated hydrophone‐based acoustic output characterization was not performed in this pilot study, MI, I_SPTA, and TI are not reported here and compliance with regulatory output limits cannot be confirmed at this stage. Nevertheless, the operating conditions were selected to be conservative relative to commonly cited diagnostic ultrasound guidance (e.g., MI ≤ 1.9 and I_SPTA ≤ 720 mW/cm^2^ for non‐fetal peripheral applications). A full hydrophone‐based characterization of MI, I_SPTA, and TI in accordance with IEC 60601‐2‐37 will be conducted in future work.

### 4.6 Design of Machine Learning Algorithms

The machine learning pipeline began with feature preprocessing. Echo‐derived parameters, including time‐of‐flight traces, amplitude ratios, and envelope energies in selected frequency bands, were extracted and assembled into structured datasets. Missing values, when present, were handled by listwise deletion to avoid introducing imputation‐related bias in this small dataset, and all features were standardized to zero mean and unit variance to eliminate scale disparities. The dataset was partitioned into training and testing subsets using a leave‐one‐subject‐out cross‐validation strategy, thereby ensuring that the model was evaluated on participants not included during training and preventing information leakage.

We implemented three regression models for bladder volume estimation: linear regression, random forest, and Extreme Gradient Boosting (XGBoost). All models were trained using the same feature set and training–testing protocol. Hyperparameters, including the number of trees, maximum depth, and minimum leaf size for the random forest, were optimized using grid search within the leave‐one‐subject‐out framework. The regression performance metrics and statistical procedures used to quantify model accuracy and agreement with the impedance‐based reference device, including MAE, RMSE, R^2^, confidence interval estimation, and Bland–Altman analysis, are described in detail in the “Statistical Analysis” subsection. Based on these criteria, the random forest regressor was selected as the final model, and its comparative performance relative to the other models is reported in Section 2.4 and Figure S12.

### 4.7 Validation and Evaluation of the Wearable Ultrasound Device

The performance of the wearable bladder ultrasound system was preliminarily evaluated in a pilot study conducted under clinically relevant conditions. A total of six healthy adult volunteers (n = 6; 3 males and 3 females; age 21–68 years) with no history of lower urinary tract disease, prior pelvic surgery, or neurological disorders affecting bladder function were recruited. All participants provided written informed consent, and the study protocol was approved by the Institutional Review Board of Qingdao University (QDU‐HEC‐2024463).

Each study session followed a standardized bladder filling protocol. At the beginning of the session, participants were instructed to void completely to establish an empty‐bladder baseline. The wearable ultrasound patch was then affixed to the suprapubic region of the lower abdomen after skin preparation with alcohol wipes, and its position was secured with a medical adhesive film. Bladder filling was induced by supervised oral intake of water at a comfortable rate. During the filling phase, paired measurements were acquired at regular intervals (every 10 min). Data collection continued until the participant reported a strong desire to void or a predefined safety limit was reached. The resulting dataset comprised 120 paired measurements across the six participants and was used both to quantify agreement between the wearable system and the impedance‐based reference device and to develop and evaluate the machine‐learning models described in Section 2.4.

At each time point, one acquisition frame was recorded by the wearable device, and a reference bladder‐volume estimate was obtained using a benchtop electrical impedance‐based measurement system (electrode‐based). For machine‐learning analysis, each recorded acquisition frame (a fixed‐duration echo trace) was partitioned into three consecutive, non‐overlapping windows of equal length spanning the full trace from start to end, yielding 360 window‐level samples derived from the 120 paired acquisitions. The benchtop electrical impedance‐based measurement system was operated by a trained researcher following a standardized measurement procedure with consistent electrode placement, and the instrument‐reported volume estimate was recorded as the reference value at each time point. Repeated reference measurements at the same time point were not performed in this pilot study; therefore, test–retest repeatability was not assessed. Formal operator blinding was not implemented in this pilot study; however, the wearable system did not display real‐time volume estimates during data collection, and all model‐based predictions were generated offline after the study. Therefore, the reference measurements were recorded without access to the wearable predicted volumes.

### 4.8 Statistical Analysis

Statistical analysis focused on evaluating regression performance and agreement between the wearable system and the impedance‐based reference device. Agreement between bladder volumes estimated by the wearable device and reference volumes obtained from the impedance‐based reference device was assessed using Bland–Altman analysis on acquisition‐level paired estimates (n = 120), including estimation of the mean bias and the 95% limits of agreement. For model inputs, echo‐derived features were standardized to zero mean and unit variance based on the training data within each leave‐one‐subject‐out fold. Missing values, when present, were handled by listwise deletion. Continuous variables are reported as summary statistics where applicable, and key performance results are reported together with 95% confidence intervals (CIs) estimated using bootstrap resampling. Model performance metrics (MAE, RMSE, and R^2^) were computed at the acquisition level (n = 120) by averaging the three window‐level predictions obtained from the same acquisition time point to yield a single volume estimate per paired acquisition. Residual analyses in Figure 4c,d are shown at the window level (n = 360; 120 × 3) to illustrate the distribution of prediction errors across all inference samples. No hypothesis testing for group differences was performed in this pilot study; therefore, no *p*‐values are reported. All analyses were performed using custom scripts in Python (version 3.12) with the scikit‐learn library and standard scientific computing packages.

## Conflicts of Interest

The authors declare no conflicts of interest.

## Supporting information




**Supporting File**: advs73834‐sup‐0001‐SuppMat.docx.

## Data Availability

The data that support the findings of this study are available in the supplementary material of this article.
