# Supplementary material for: Integrated Ultrasound Device for Precision Bladder Volume Monitoring via Acoustic Focusing and Machine Learning
Source: Adv Sci (Weinh). 2026 Jan 20;13(17):e20926. doi: 10.1002/advs.202520926 (PMC13042915; doi:10.1002/advs.202520926)
Supplement: Supplementary file 1 — Supporting File: advs73834‐sup‐0001‐SuppMat.docx. [file ADVS-13-e20926-s001.docx]

Supporting Information

**Integrated Ultrasound Device for Precision Bladder Volume Monitoring via Acoustic Focusing and Machine Learning**

*Long Long Cao, Feng Wen Wang, Jingwei Xue, Fulei Liu, Ming Liang Jin**


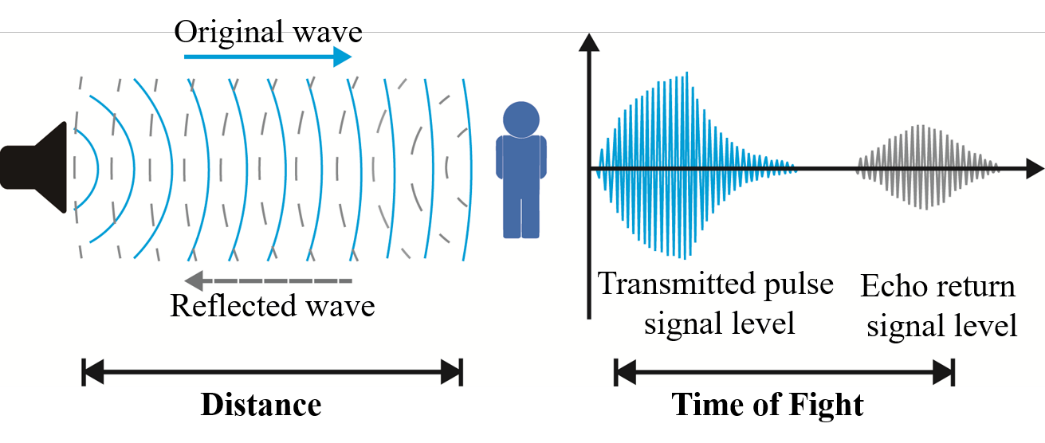


Figure S1. Principle of ultrasonic distance measurement.


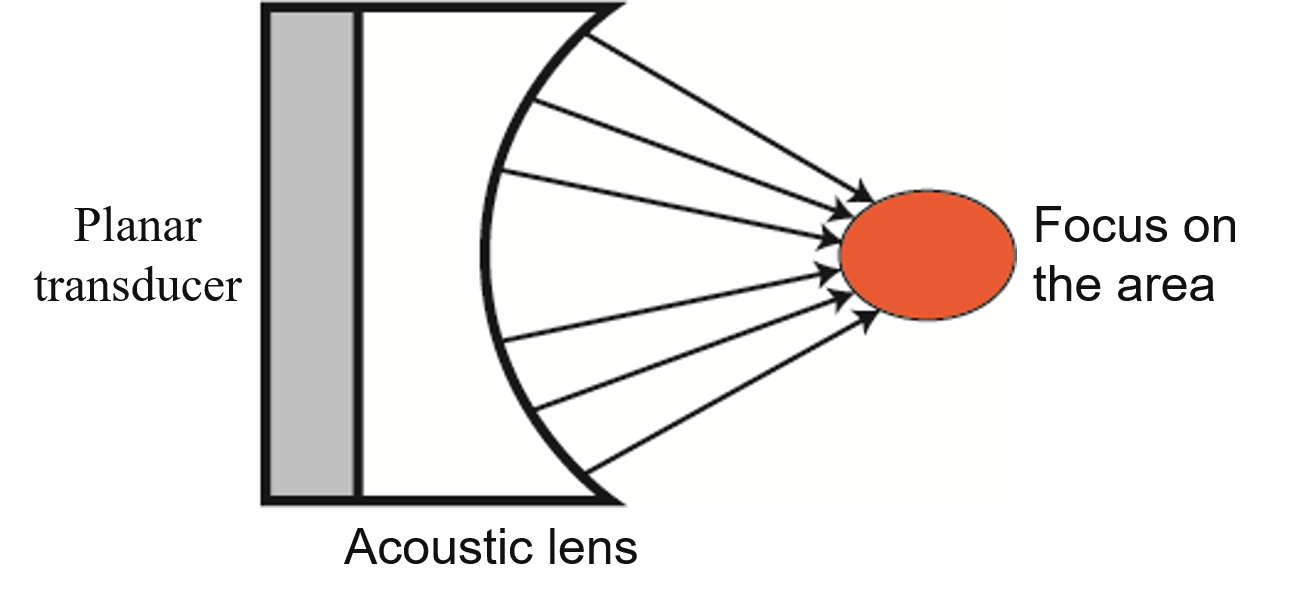


**Figure S2**. Schematic diagram of the single-sided acoustic lens focusing principle.


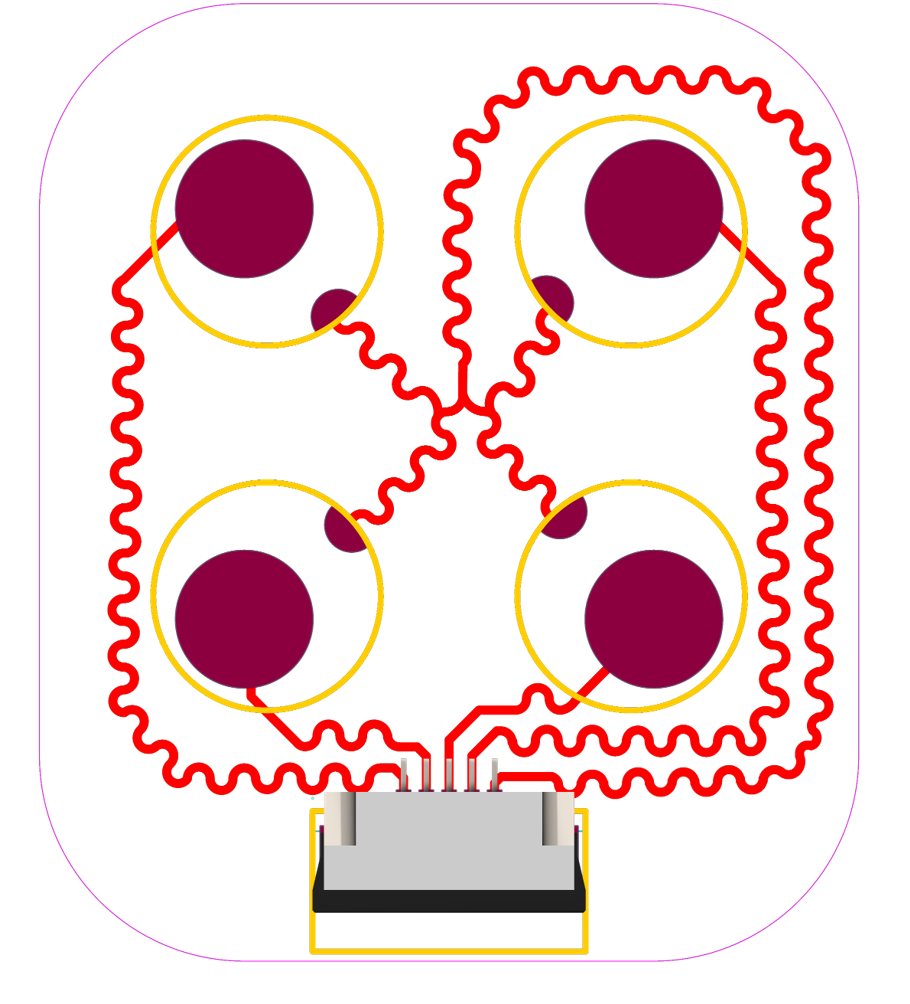


**Figure S3**. Serpentine circuit design for ultrasonic transducer arrays.


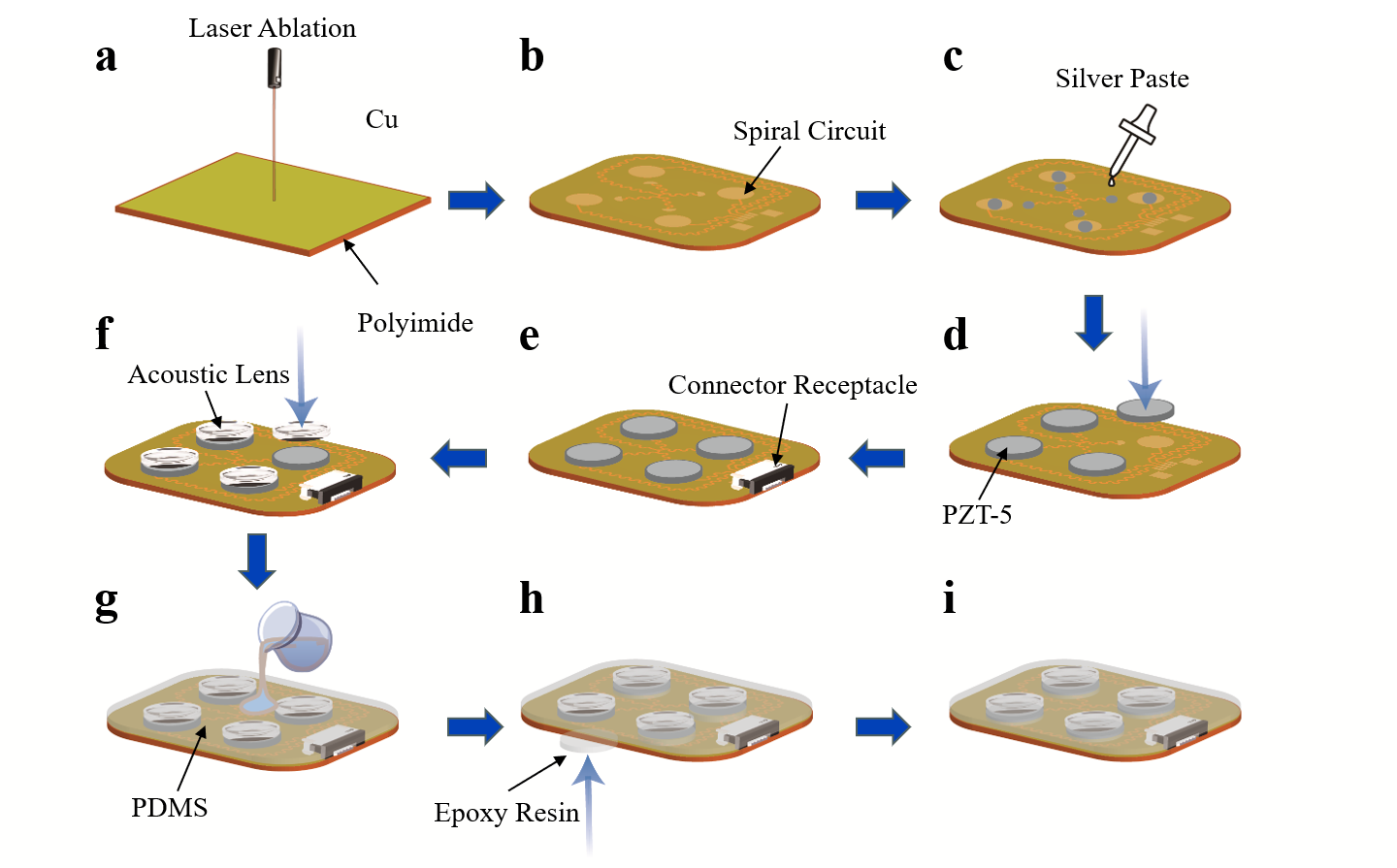


**Figure S4**. The fabrication process of the ultrasonic transducer array.


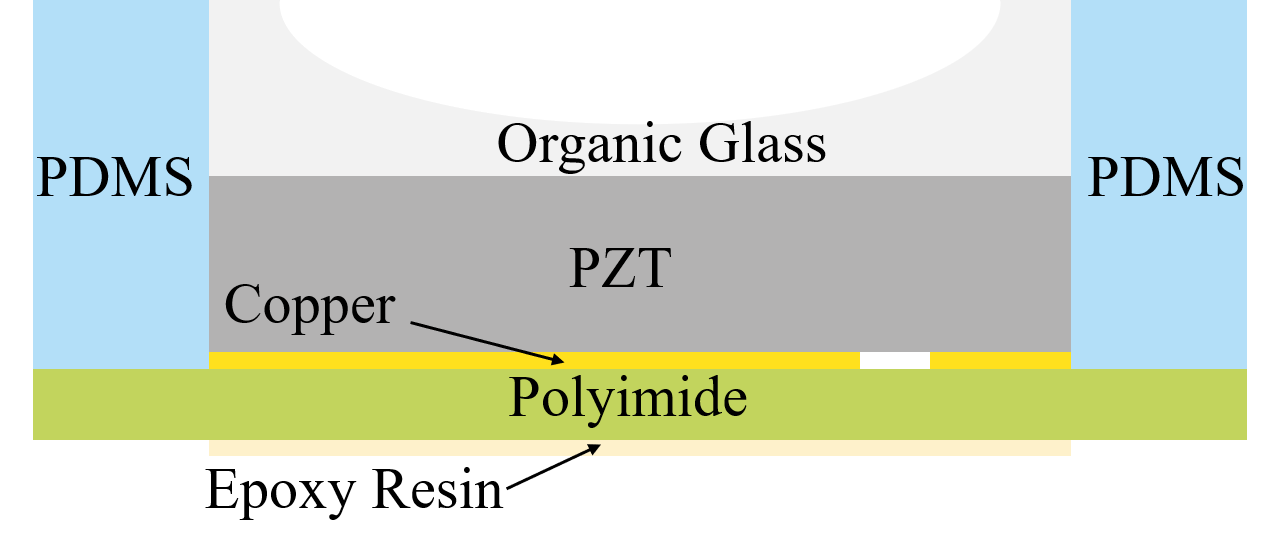


**Figure S5**. The cross-section of the flexible ultrasonic probe.


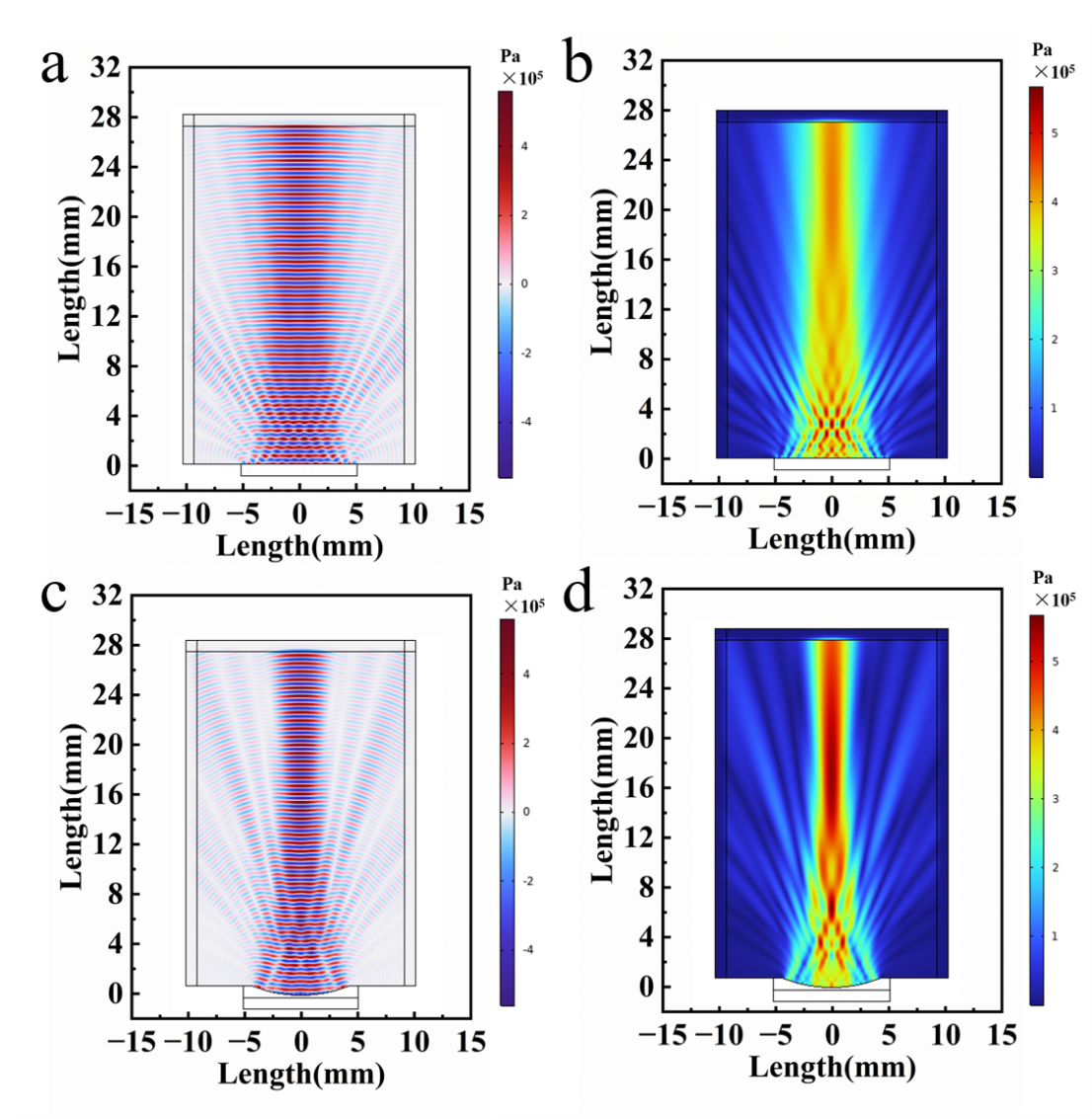


**Figure S6**. Comparison of Simulated Acoustic Fields with and without the Lens. (a)Simulated acoustic pressure distribution without the lens; (b) Simulated total acoustic field without the lens; (c) Simulated acoustic pressure distribution with the lens; (d) Simulated total acoustic field with the lens.


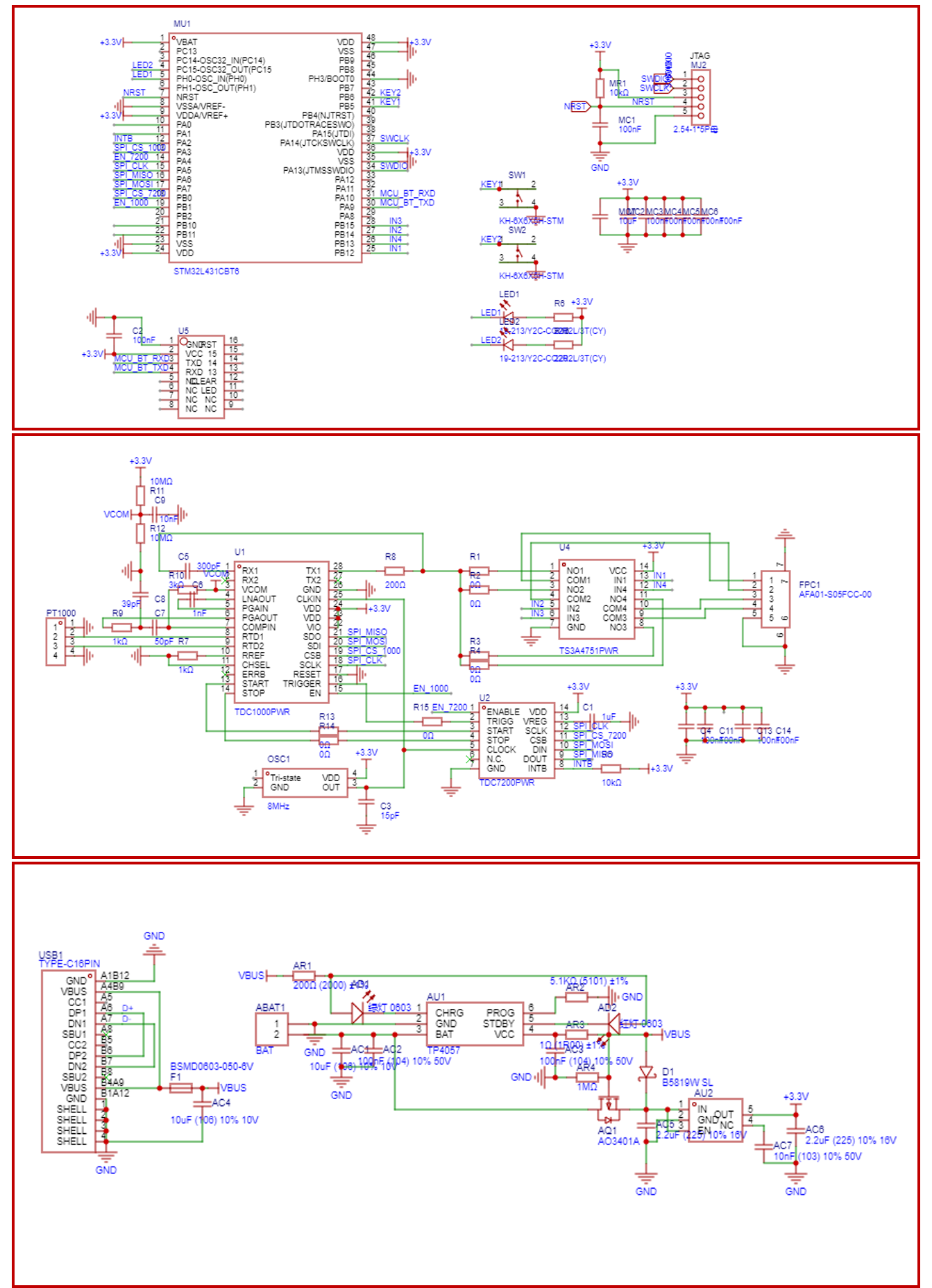


**Figure S7**. Schematic circuit diagram of the TDC1000/TDC7200-based wearable ultrasound system with a TS3A4751 4-channel analog switch for multiplexing the 2×2 array.


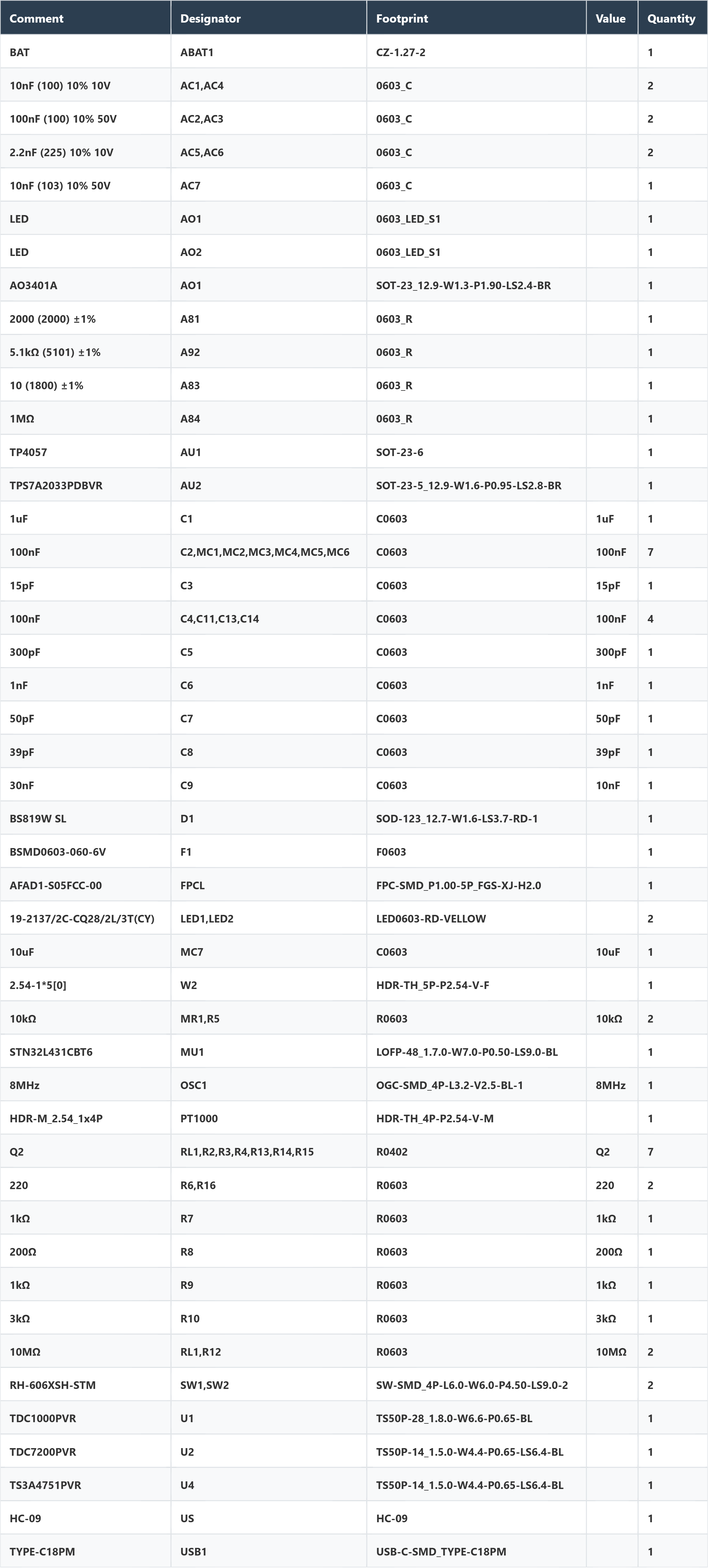


**Figure S8**. Electronic component list.


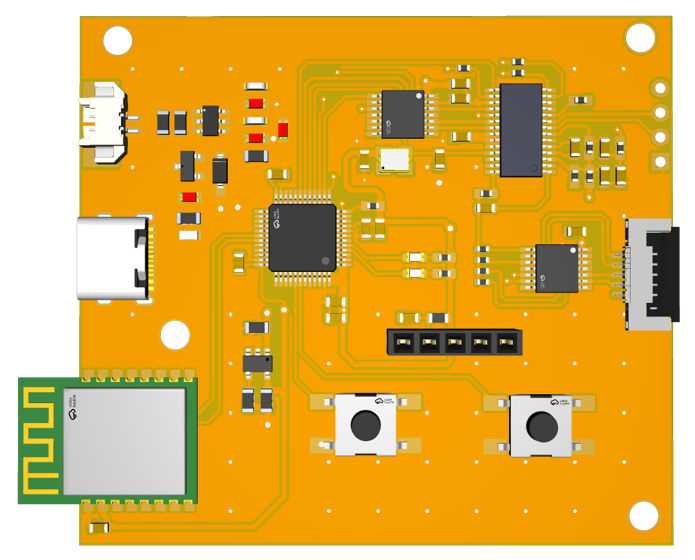


**Figure S9**. Top layer of the circuit board.


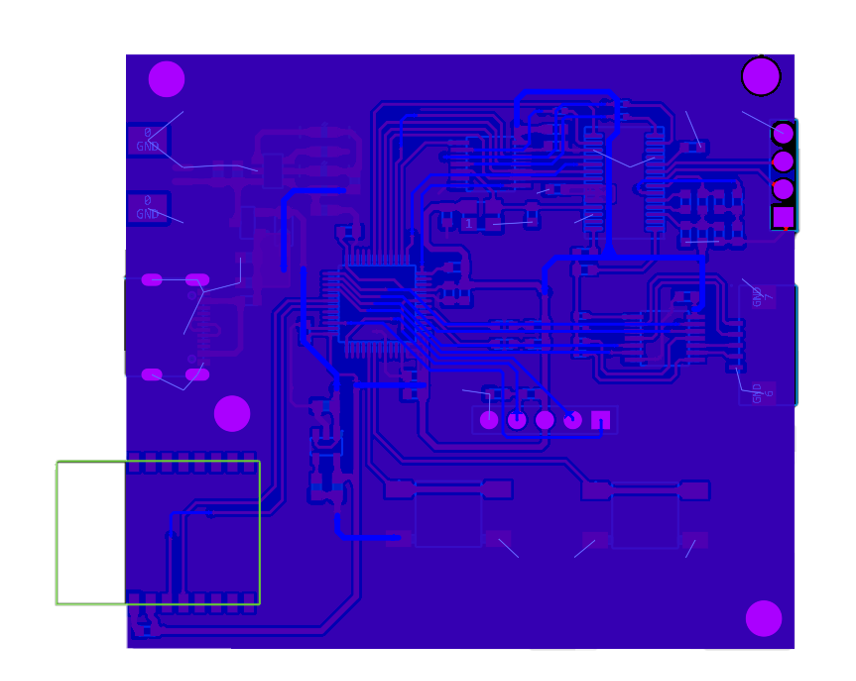


**Figure S10**. The bottom layer of the circuit board.


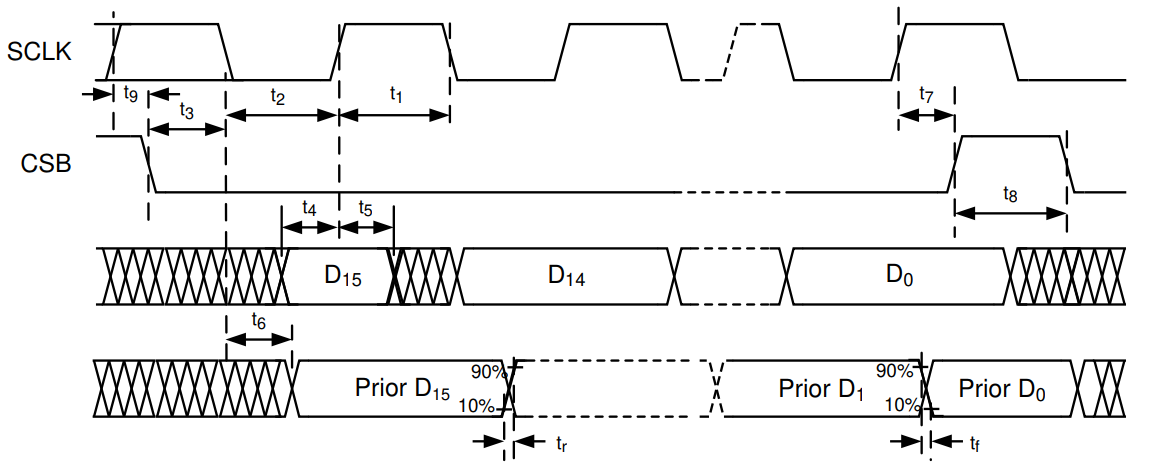


**Figure S11**. SPI Timing Diagram.


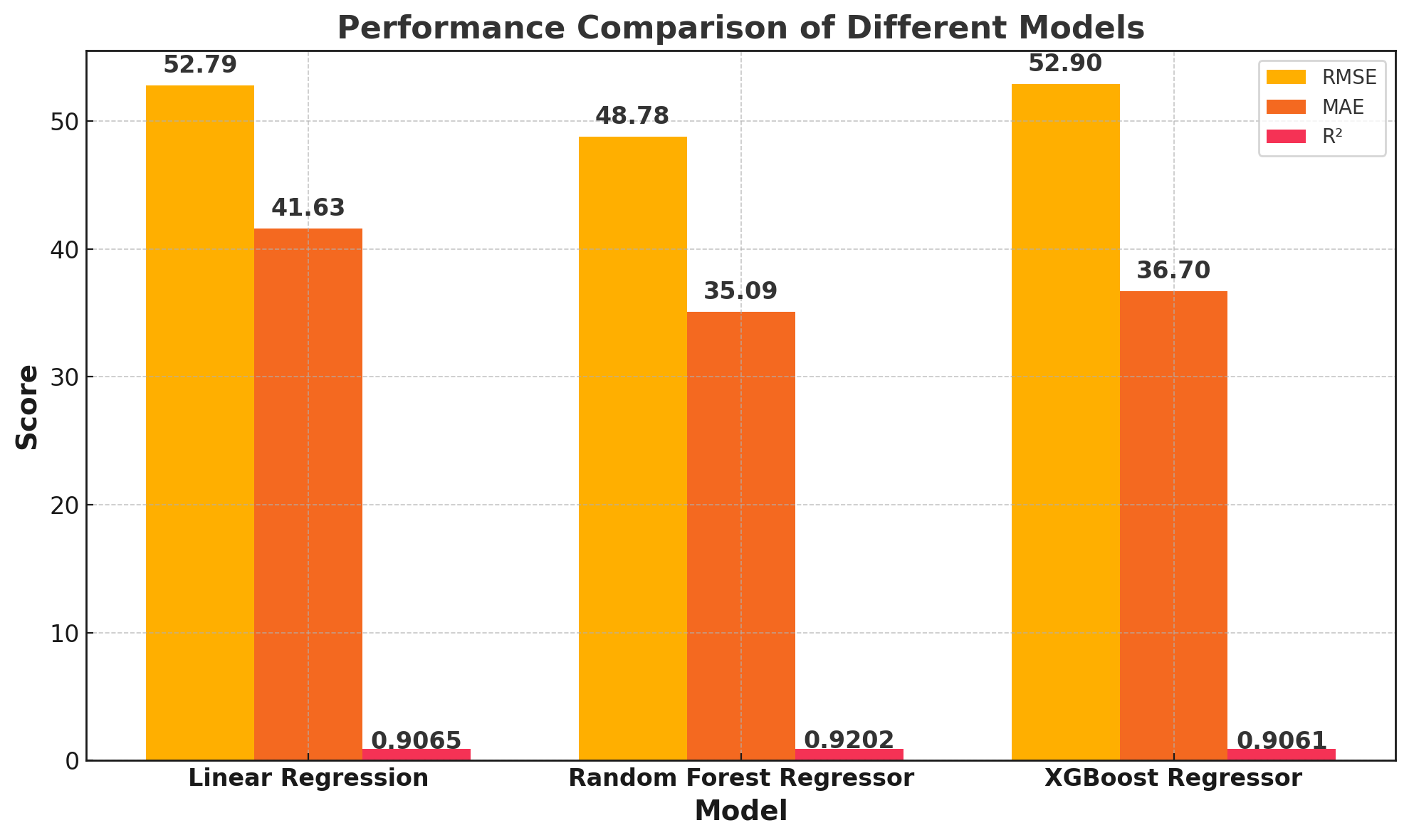


**Figure S12**. Model Performance Comparison: RMSE, MAE, and R². Model comparison was performed using leave-one-subject-out cross-validation on n = 120 paired measurements from 6 participants.

**Table S1.** Comparison between the proposed device and representative bladder/wearable ultrasound systems.

|  | **Target application** | **Front-end driver** | **Acquisition** | **Multiplexing** | **MCU** | **Data processing** |
| --- | --- | --- | --- | --- | --- | --- |
| This work | Bladder volume | Integrated ultrasonic AFE (TDC1000) | Echo detection via TDC1000 and TOF measurement by TDC7200 | 2×2 array TDM via TS3A4751 | STM32L431 | Machine-learning regression (volume estimation) |
| Ref. 17 | Tumor therapy | Custom high-voltage driver (for therapeutic HIFU excitation) | N/A (therapy-oriented) | Driver network | On-board controller with wireless interface | Therapy parameter control |
| Ref. 20 | Central BP | Bench-top pulser (5077PR, Olympus) | Echo by oscilloscope | None | External instruments | TOF-based arterial waveform analysis |
| Ref. 31 | Bladder volume | High-voltage pulser (MAX14808) | OPA357 and MAX941 | DG408/DG409 analog multiplexers | STM32WB55 | Geometric model for bladder volume estimation |

***Note:***

Ref. 17 F. Zou, Y. Luo, W. Zhuang, T. Xu, *Advanced Materials* **2024**, *36* (39), 2409528.

Ref. 20 C. Wang, X. Li, H. Hu, L. Zhang, Z. Huang, M. Lin, Z. Zhang, Z. Yin, B. Huang, H. Gong, *Nature biomedical engineering* **2018**, *2* (9), 687.

Ref. 31A. T. Toymus, U. C. Yener, E. Bardakci, Ö. D. Temel, E. Koseoglu, D. Akcoren, B. Eminoglu, M. Ali, R. Kilic, T. Tarcan, *Nature Communications* **2024**, *15* (1), 7216.
